# Can Informativity Effects Be Predictability Effects in Disguise?

**DOI:** 10.3390/e27070739

**Published:** 2025-07-10

**Authors:** Vsevolod Kapatsinski

**Affiliations:** Department of Linguistics, University of Oregon, Eugene, OR 97403-1290, USA; vkapatsi@uoregon.edu

**Keywords:** informativity, predictability, reduction, sound change, phonetics, usage-based linguistics, corpus linguistics

## Abstract

Recent work in corpus linguistics has observed that informativity predicts articulatory reduction of a linguistic unit above and beyond the unit’s predictability in the local context, i.e., the unit’s probability given the current context. Informativity of a unit is the inverse of average (log-scaled) predictability and corresponds to its information content. Research in the field has interpreted effects of informativity as speakers being sensitive to the information content of a unit in deciding how much effort to put into pronouncing it or as accumulation of memories of pronunciation details in long-term memory representations. However, average predictability can improve the estimate of local predictability of a unit above and beyond the observed predictability in that context, especially when that context is rare. Therefore, informativity can contribute to explaining variance in a dependent variable like reduction above and beyond local predictability simply because informativity improves the (inherently noisy) estimate of local predictability. This paper shows how to estimate the proportion of an observed informativity effect that is likely to be artifactual, due entirely to informativity improving the estimates of predictability, via simulation. The proposed simulation approach can be used to investigate whether an effect of informativity is likely to be real, under the assumption that corpus probabilities are an unbiased estimate of probabilities driving reduction behavior, and how much of it is likely to be due to noise in predictability estimates, in any real dataset.

## 1. Introduction

Research in corpus linguistics has found that the informativity of a word predicts its degree of articulatory reduction above and beyond the unit’s predictability given the current context [1,2,3,4,5,6,7,8,9,10,11]. These findings have been interpreted as evidence for speakers being sensitive to a word’s information content in deciding how to pronounce it and for long-term memory representations of words accumulating information about its predictability or pronunciation details or both [1,2,3,4,5,6,7,8,9,10,11,12,13,14,15,16,17,18,19,20,21,22,23]. The effects of informativity are remarkably consistent in that all studies that have found an effect of local predictability in a model that included informativity also found an effect of informativity, suggesting that whenever speakers are sensitive to the probability of a word given a particular context, this information also accumulates in memory in association with that word. Consequently, these effects have been argued to provide support for “rich memory” views of the mental lexicon, in which every episode of producing or perceiving a word leaves a trace [13,14,15,16,17,18,19,20,21,22,23].

However, the predictability of a word in a context is not independent of its average predictability across contexts, which is the inverse of informativity and is therefore perfectly correlated with it [24]. Furthermore, when a context is rare, the word’s predictability given that context is unreliable and can be improved by taking the word’s average predictability into account. To take an extreme example, suppose that the word *inched* occurs only once in the corpus. Then all words following it would have a probability of zero, except for the word that happened to occur after it (let us say *closer*), which would have a probability of 1. These estimates are highly unreliable, since they are based on a sample size of 1. By taking into account probabilities of words in other contexts, we might be able to estimate, for example, that *up* is a lot more probable than *higher* following *inched*. This is especially helpful for a bigram like this, where the words being predicted (*up* and *higher*) are frequent enough to frequently occur in other contexts where their predictabilities can be computed, and the present context (*inched*) is rare so predictability given the current context is unreliable. If instead we have a rare word in a frequent context that co-occurs with it, e.g., *by Kapatsinski*, then informativity will not be of much help as the predictability given the current context is reliable and informativity likely aligns with it. At the limit, if the target word only occurs in the current context, its informativity is its predictability times minus one.

In computational linguistics, this idea is behind the technique of using back-off, where the next word is predicted from a long preceding context but if this context is absent from the training data or less frequent than a predetermined cutoff, the model backs off to using a shorter context, as first proposed in [25]. Jaeger [26] has shown that the estimated effect of predictability on reduction increases when predictability estimates are improved by using back-off.

In inferential statistics, this observation is behind the idea of hierarchical/mixed-effects models, which make use of adaptive partial pooling of evidence [27]. Hierarchical regression is preferrable to back-off because it does not use an arbitrary frequency cutoff above which only local context is used and below which it is not used at all. The cutoff falsely assumes that there is zero information (i.e., only noise) in contextual predictability when the context frequency is below the cutoff and that out-of-context probabilities cannot improve in-context probability when context frequency is above the cutoff (for empirical evidence that these assumptions are false, see [28,29]). Ideally, one would take into account both in-context and out-of-context probabilities at all times but optimally weigh them based on how reliable they are in each context. This is what adaptive partial pooling is designed to do. Computational linguistics approaches that have replaced n-gram models with back-off, like recurrent networks and transformers, also implicitly make use of partial pooling. We return to this point in the Discussion.

In estimating some variable in some context, partial pooling relies on observations from that context to the extent that they are available, because they are more relevant than observations from other contexts. But the fewer such observations that are available, the more the model’s estimate is based on observations from other contexts. It can be shown that the estimate resulting from partial pooling is generally more accurate than an estimate that does not take other contexts into account (for simulations in the context of estimating reduction probabilities, see [28]). In corpus data, context frequency is 1 for a large proportion of contexts. For example, if context is operationalized as the preceding or following word, as in [1], context frequency would be 1 for ~50% of contexts in any corpus, as shown in [30]. Therefore, partial pooling would yield substantial benefits in estimating predictability from naturalistic language input compared to relying completely on observed predictability in a given context. If speakers estimate predictability in context, it would therefore behoove them to rely on partial pooling to do so [17].

How does partial pooling work? In hierarchical regression [27], the partial pooling estimate for some characteristic ($\hat{\alpha }$) of a word (*w*) in a context (*cx*) would be estimated by Equation (1):

(1) $${\hat{\alpha }}_{w|cx}=\frac{\frac{n_{cx}}{\sigma_{cx}^{2}}}{\frac{n_{cx}}{\sigma_{cx}^{2}}+\frac{1}{\sigma_{w}^{2}}}\alpha_{w|cx}+\frac{\frac{1}{\sigma_{w}^{2}}}{\frac{n_{cx}}{\sigma_{cx}^{2}}+\frac{1}{\sigma_{w}^{2}}}\alpha_{w}$$

Here, $n_{cx}$ is the number of observations (frequency) of the context *cx*, $\sigma_{cx}^{2}$ is the observed variance of the characteristic (α) across words in the context *cx*, and $\sigma_{w}^{2}$ is the variance of average $\alpha_{w}$ across words ($\alpha_{w}$ being averaged across all contexts in which each word occurs). Equation (1) says that the estimate based on the current context ($\alpha_{cx}$, here predictability) is weighted more when the context is frequent ($n_{cx}$ is high), and words tend not to vary in α within that context. Conversely, the estimate based on other contexts (informativity) is weighted more when words (within the relevant class) tend not to differ in (α). If $\alpha_{w|cx}$ is predictability of the word given context, the variance parameters are likely to be relatively unimportant because word predictability distributions within and across contexts are (approximately) scale-free with an exponent of ~1, i.e., Zipfian [30,31], so $\sigma_{w}^{2}$ and $\sigma_{cx}^{2}$ tend not to vary dramatically, while $n_{cx}$, which also follows a scale-free Zipfian distribution, varies enormously across contexts. Therefore, the main influence on the importance of observed local predictability ($\alpha_{w|cx}$) vs. observed informativity ($\alpha_{w}$) in estimating local predictability (${\hat{\alpha }}_{w|cx}$) given a context should be the frequency of that context.

The present paper demonstrates that, as expected from the reasoning above, the apparent effect of informativity is larger in rare contexts than in frequent ones, while the effect of local, contextual predictability is smaller in rare contexts. This follows directly from the fact that contextual predictability estimates are less reliable in rare contexts and that informativity can help improve these estimates via partial pooling.

Previous simulation work on this question by Cohen Priva and Jaeger [24] has investigated whether the *segmental* informativity effect observed in [32] could be spurious, concluding that it is highly unlikely to be. However, this work did not try to simulate the effects of predictability and informativity on an observable dependent variable like duration, instead asking how correlated informativity is with predictability and vice versa as a function of sample size. This does not allow one to determine how large spurious effects of the two correlated variables on a dependent variable would be. Because the dependent variable and the sizes of the effects of informativity and predictability were not simulated, [24] also does not allow a researcher to use the simulation approach to infer whether an effect of informativity they have observed is attributable to predictability.

The present paper extends this simulation approach by modeling the dependent variable. In addition, it extends it by modeling lexical rather than segmental predictability and informativity. In [24], Cohen Priva and Jaeger mention conducting simulations of informativity and predictability at the lexical level in the discussion section but do not describe the results beyond stating that the “results of those additional studies were similar, except that the problems observed here were observed more strongly and even for larger lexical databases when words, instead of segments, were analyzed. This is presumably due to the overall much larger number of word types, compared to segment types. For example, spurious frequency effects were found when predicting word predictability or word informativity, even for lexical databases with 10,000,000 words” (p.11). As noted in this quote, sampling noise is far less of a concern on the segmental level than on the lexical level, because even the rarest segments like [ð] or [ʒ] in English are very frequent relative to the average word. At the same time, most studies that have argued for an effect of informativity have been conducted at the lexical level [1,2,3,4,5,6,7,8,9,10,11]. Given that spurious effects are more likely at the lexical level, it is important to have a methodology to determine whether any particular lexical informativity effect might be a predictability effect in disguise.

We show, by simulation, that a significant effect of informativity and its greater magnitude in rare contexts could sometimes be due to sampling noise in the predictability estimates. Furthermore, we provide a simple simulation approach to estimate *how much* of an observed effect of informativity is spurious, i.e., due to sampling noise. For our actual example, much of the effect of informativity is not due to sampling noise in predictability estimates. That is, although corpus data could show a significant effect of informativity in the same direction even if speakers showed no real sensitivity to informativity, our simulations show that the magnitude of that spurious effect would rarely be as large as the effect in the real data.

## 2. Materials and Methods

We begin by demonstrating that predictability and informativity behave as one would expect if (some of) the effect of informativity were due to partial pooling, i.e., informativity helping reduce noise in predictability estimates. Because there is more noise in smaller samples, this involves showing that these predictors interact with context frequency, such that the effect of predictability is stronger in frequent contexts and the effect of informativity is stronger in rare ones. Previous work in [26] has shown that the effect of predictability is stronger in frequent contexts but has not examined whether the effect of informativity interacts with context frequency.

The data we model come from the Switchboard Corpus [33], using the revised word-to-speech alignments from [34]. To ensure that the results generalize across the informativity and predictability continua, I selected two datasets that are opposite of each other in terms of informativity and predictability. One dataset (*n* = 5146) consists of the words that followed repetition disfluencies in [35], which are mostly nouns. These words are relatively high in informativity and low in predictability, as these factors predict the occurrence of a disfluency before a word [35]. The other dataset (*n* = 5187), helpfully provided by T. F. Jaeger, consists of determiners (*the*, *a*, *an*, *that*, *this*, *these*, *those*, *no*, *every*, *some*, *each*, *another*, *both*, *any*, *all*, *either*, *one*, *many*, *neither*, or *half*) in prepositional phrases, extracted from the Penn Treebank II Switchboard corpus. None of the determiners are adjacent to disfluencies or pauses. These words are relatively low in informativity and high in predictability. In both datasets, context was operationalized as the preceding word in the utterance, so predictability is $log(p\left(w_{n}|w_{n-1}\right))$, i.e., forward bigram surprisal with a flipped sign, and informativity is surprisal averaged across contexts.

For the post-disfluency dataset, LSTM [36] probabilities are also available, as described in [35]. However, bigram surprisal is preferrable to surprisal from neural models like [36] for the present purpose of comparing predictability and informativity because part of the power of neural models is that they implicitly implement adaptive partial pooling, relying largely on the specific local context when it is familiar and generalizing over similar contexts when the context is new [37]. Therefore, surprisal/predictability estimates from neural models are influenced by informativity through partial pooling to an unknown extent, since we do not have the equivalent of Equation (1) for neural models. Furthermore, the bigram operationalization makes it easier to determine which two contexts are “the same” for the purposes of calculating context frequency. That is, words that end in the same word are “the same context” for the present study.

Both datasets contain word tokens that are relatively controlled for other factors that influence durations. For the post-disfluency dataset, they are mostly nouns following high-frequency function words (prepositions or articles) [35,38], and surrounding words outside of the disfluency itself have little influence on their durations [38]. This set of words is also of theoretical interest because there is no previous data investigating whether the durations of words following a disfluency are influenced by predictability, and one might expect the interruption in the flow of speech constituting the disfluency to reset the context for speech planning so that predictability given the word preceding the interruption would not influence duration of the word following the interruption. Below, we show that this is not the case. The determiner dataset is controlled for local syntactic and prosodic context: all determiners are in prepositional phrases, and the dataset contains annotation for prosodic word boundaries. All tokens with adjacent boundaries were excluded to make the dataset maximally different from the other.

The two maximally different datasets are intended to illustrate that the choice of the dataset matters little, as nothing in the argument hinges on the choice of the dataset. The predictions follow simply from the mathematical relationship between predictability and informativity, which ensures that the interactions with context will arise as a side effect of the Law of Large Numbers. Thus, these interactions should always be observed given enough power. The simulations below make no assumptions about what the data distribution looks like and bootstrap it from whatever data the researcher is hoping to simulate. For example, this methodology has also been applied to word durations in a Polish corpus, based on guidance from the present author serving as a reviewer (see [11]). Furthermore, the interaction between predictability and context frequency was also seen in [26].

We fit a regression model, predicting word duration (log scaled to reduce skew, as is standard in the literature) from predictability, informativity and their interactions with context. We fit a simple least squares linear regression model (Table 1), using lm() in base R [39], and a mixed-effects lmer() model using the lme4 package [40], version 1.1-35.5, with random intercepts for target words whose durations we model. The random intercepts allow us to control for uncontrolled variables that vary across words along with informativity and thus might spuriously strengthen or weaken the informativity effect independently of its relationship with predictability. I also tried adding a random slope for predictability by word. This model did not converge in the post-disfluency dataset because 64% of the words in the sample occur only once and thus cannot be used to test the random slope. Therefore, the random-effects structure for that dataset consists only of the random intercept for word. The random-effects structure for the determiners model in Table 2 is maximal: it also includes a random slope for predictability and the correlation between the two. *p* values for linear mixed-effects models were generated using Satterthwaite’s method for approximating degrees of freedom for *t* tests in mixed-effects models [41]. Note that, because the random-effects structure is more complex for determiners, there are more degrees of freedom in the post-disfluency model for the same *n*. Therefore, the same *t* value corresponds to a lower *p* value in the post-disfluency model compared to the determiner model. For the models with interactions in Table 3, the most complex random-effects structure that converged for both datasets included only random intercepts, thus random slopes were not included. Marginal and conditional *R*^2^ values for the mixed-effects models were calculated using the r.squared_GLMM() function in the MuMIn package [42].

To simulate how much of the effect of informativity might be due to predictability, we repeatedly generate data samples in which predictability has the bivariate relationship with duration observed in the real data while informativity has no effect, i.e., the variance not accounted for by predictability is random observation-level noise (if the data are generated from a fixed-effects-only model) or observation-level noise plus the effects of lexical identity (estimated from the observed real data by the mixed-effects model with predictability as a fixed effect and word identity as a random intercept). The true magnitude of the predictability effect is constant across contexts, regardless of context frequency. We then fit the same model we fit to the real data, with predictability and informativity as predictors, with or without context frequency and interactions with it. We ask: (1) how often do we get a “false alarm” on informativity: how often do we observe a significant effect of informativity (at *p* < 0.05) in the same direction as in the real data, even though the data are generated by a process in which there is no true effect of informativity, and (2) how often is that effect at least as large as the effect in the real data.

Specifically, the probability of each word given each context, $p(w_{n}|w_{n-1})$, feeds into a binomial distribution with the generated context frequencies serving as the number of “trials” in each context. For each “trial”, i.e., token of the preceding word, a biased coin is tossed, which comes up heads with the probability given by $w_{n}$’s probability given the preceding word in the actual sample, $p(w_{n}|w_{n-1})$. I have also tried varying context frequencies across simulations by sampling context tokens from a multinomial distribution fit to the probabilities in the sample, but this does not appear to change the likelihood of observing a spurious effect of informativity compared to simply using the observed frequencies, thus the simpler approach is taken here.

The binomial sampling process inevitably produces sampling noise. For example, if you toss a coin that has a 50% probability of coming up heads 5 times, the coin will not come up heads on exactly 2.5 occasions and sometimes will come up heads in all 5 trials 3% of the time. The effect of this sampling noise is what adding informativity to the regression model can alleviate. Furthermore, the fewer times you toss the coin, i.e., the lower the context frequency, the more variable probabilities across coin tosses will be given that the underlying probability of the coin coming up heads is kept the same. For example, if you toss the coin once, the coin will generate a probability of 0 or 1 in each sample regardless of underlying probability. If you toss it 5000 times, the average will be close to the underlying probability. This is why we expect predictability effects to be stronger in frequent contexts and why we expect the spurious component of the informativity effect to be largest in rare contexts: in a rare context, estimated predictability is on average farther from the true predictability that influenced duration, and therefore more of the effect of true predictability remains to be captured by informativity.

Predictability of a word given context times the coefficient for predictability in the model without informativity plus the random effect of a word generates the mean of a normal distribution of log duration for that word in that context, and the standard deviation of the residuals of the model serves as the standard deviation of the normal. The simulated duration of each word token is then generated by sampling from this distribution.

The post-disfluency dataset, analysis and simulation code are available on OSF: https://osf.io/c8aws, accessed on 4 July 2025. I do not have permission to redistribute the determiner dataset.

## 3. Results

### 3.1. Predictability, Informativity and Context Frequency

Table 1 and Table 2 present the linear regression model results. Table 1 presents an analysis in which predictability and informativity jointly predict duration, as in previous empirical work in [1,2,3,4,5,6,7,8,9,10,11]. The two predictors together explain 42.8% of variance in duration of post-disfluency words and 48.2% of variance in duration of determiners in prepositional phrases (based on adjusted R^2^). In both datasets, the two predictors are collinear but much more so in the post-disfluency dataset (*r* = –0.91 for the post-disfluency dataset vs. *r* = –0.65 for the determiner dataset). This difference in correlation strength follows from the greater frequencies of determiners, which will often be observed outside of the current context, on which predictability is based. If a word is only observed in the current context, which is true for many post-disfluency words but not true for determiners, predictability is necessarily informativity times minus 1. In both datasets, informativity explains more variance than predictability, but especially so in the determiner dataset: removing informativity from the model in Table 1 reduced adjusted R^2^ by 3% in the post-disfluency dataset and by 23% in the determiner dataset, while removing predictability reduces it by <1% in both datasets.

**Table 1:** Linear regression results predicting log word duration (log seconds) from predictability and informativity. Adjusted *R*^2^ = 0.439 for post-disfluency words and = 0.482 for determiners.

Post-Disfluency Words	*b*	*se* (*b*)	*t*	*p*
Intercept	−2.27	0.017	−132.98	<0.00001
Predictability	−0.05	0.007	−6.89	<0.00001
Informativity	0.13	0.007	18.83	<0.00001
**Determiners in PPs**	***b***	***se* (*b*)**	***t***	***p***
Intercept	−0.05	0.002	−18.91	<0.00001
Predictability	−0.004	0.001	−6.41	<0.00001
Informativity	0.05	0.001	46.47	<0.00001

Adding the random effects to the models without interactions somewhat reduces the informativity and predictability effect magnitudes, but the qualitative pattern of results remains unchanged (Table 2): both effects are significant and the informativity effect is stronger than the predictability effect.

**Table 2:** Linear mixed-effects regression results predicting log word duration (log seconds) from predictability and informativity, as in previous work. Marginal *R*^2^ = 0.27 for post-disfluency words and = 0.31 for determiners. Conditional *R*^2^ = 0.47 for post-disfluency words and 0.61 for determiners.

Post-Disfluency Words	*b*	*se* (*b*)	*t*	*p*
Intercept	−1.81	0.03	−132.98	<0.00001
Predictability	−0.02	0.005	−4.13	=0.00004
Informativity	0.09	0.006	13.90	<0.00001
**Determiners in PPs**	***b***	***se* (*b*)**	***t***	***p***
Intercept	−0.02	0.038	−0.59	<0.00001
Predictability	−0.003	0.001	−4.20	=0.0123
Informativity	0.04	0.007	5.31	=0.00003

**Figure 1:**
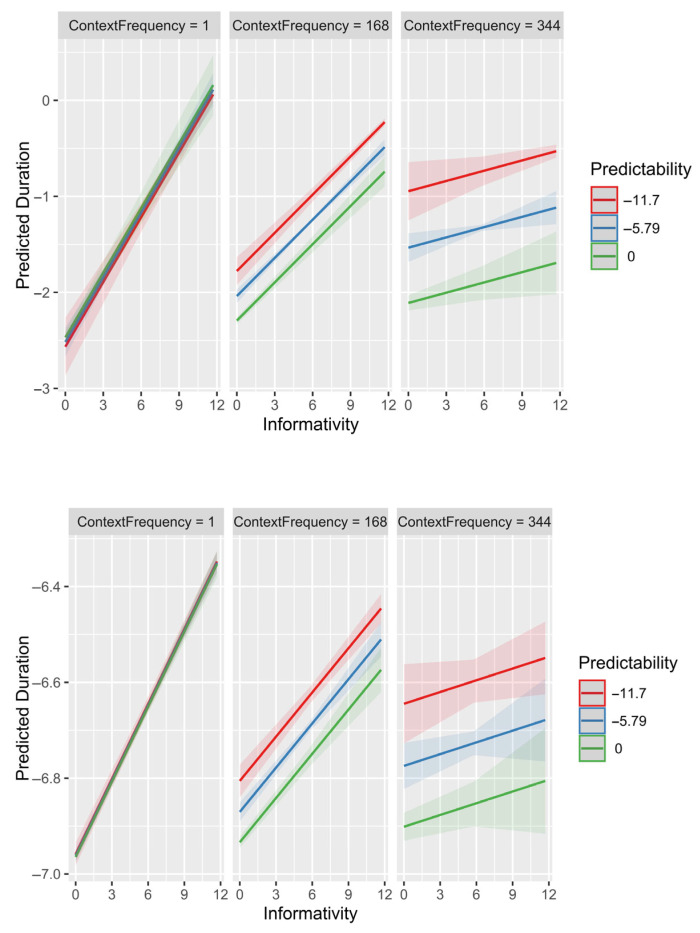
Fixed-effects-only model predictions showing interactions of predictability (x axis) and informativity (lines) with the square root of context frequency (panel). Top row: Post-disfluency words. Bottom row: Determiners in prepositional phrases. The square root is chosen because it scales linearly with standard error and therefore should be a good measure of the amount of information about predictability in each context. The three lines and three panels show the effect for the minimum, mean and maximum (in the dataset) value of each predictor in the Post-disfluency dataset. Since the interactions are linear, the lines for the intervening values would fall between the lines shown. Figure generated using the plot_model() function (type = “emm”) from the sjPlot package [43].

**Figure 2:**
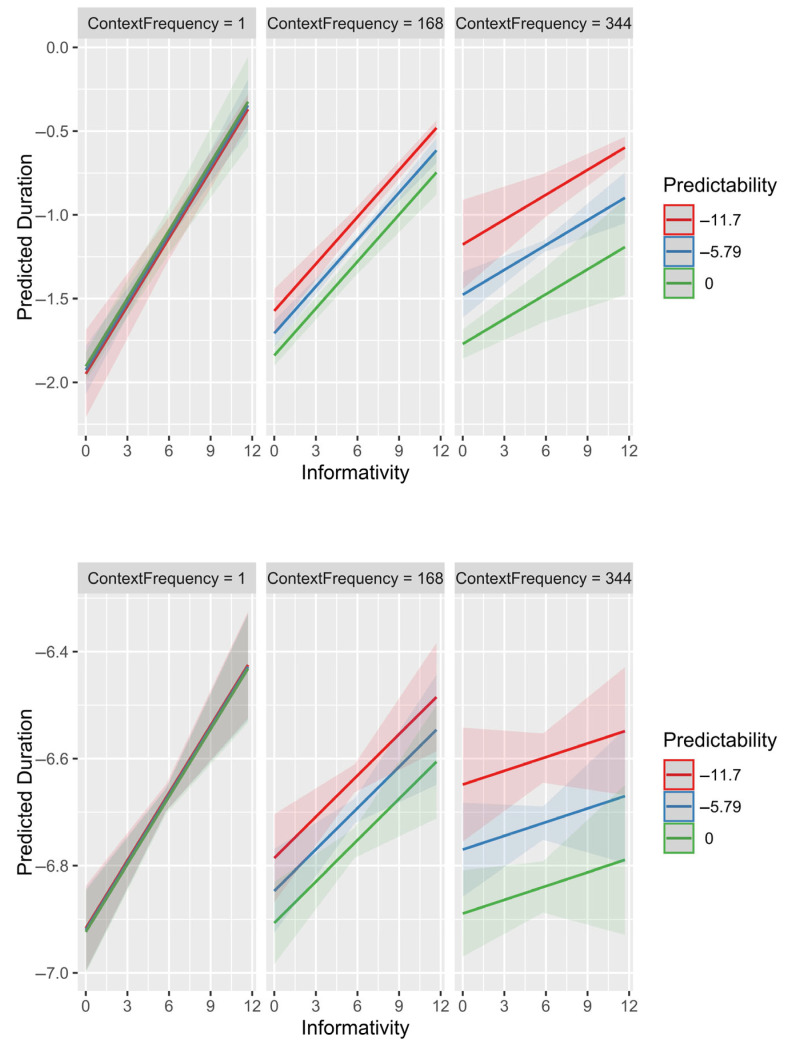
Mixed-effects model predictions showing interactions of predictability (x axis) and informativity (lines) with the square root of context frequency (panel). Top row: Post-disfluency words. Bottom row: Determiners in prepositional phrases. The three lines and three panels show the effect for the minimum, mean and maximum (in the dataset) value of each predictor in the Post-disfluency dataset.

The model in Table 3 adds interactions with context frequency predicted by partial pooling to the model in Table 2 (a model omitting random effects produced qualitatively identical results). Figure 1 and Figure 2 plot these interactions for the two datasets. Figure 1 shows the effects on durations of post-disfluency words, while Figure 2 shows the effects on durations of determiners in prepositional phrases.

Despite these word types being on the opposite ends of the informativity, predictability, frequency and duration distributions and the difference in context fluency, the interactions of predictability and informativity with context frequency are almost identical. When the context is new/observed only once in the corpus, there is no effect of predictability given the context (context frequency = 1; left panels), as seen from all the lines being on top of each other, and a strong effect of informativity (steep line slope). When the context is very frequent (right panels), there is a strong effect of predictability given that context (the lines are well separated) and a much reduced if not zero effect of informativity (line slopes are shallow and the confidence regions for determiners include a slope of 0 corresponding to no effect of informativity).

**Table 3:** Linear mixed-effects regression results predicting log word duration (log seconds) from predictability, informativity and their interactions with context (preceding word) frequency. Adjusted *R*^2^ = 0.448.

Post-Disfluency Words	*b*	*se* (*b*)	*t*	*df*	*p*
Intercept	−1.91	0.043	−44.53	2577.2	<0.00001
Context Frequency	0.0004	0.00002	2.20	5033.6	0.028
Predictability	0.004	0.011	0.36	4434.3	0.72
Informativity	0.136	0.013	10.59	5076.0	<0.00001
Predictability:Context Frequency	−0.0002	0.0001	−2.63	4698.3	0.00864
Informativity:Context Frequency	−0.0003	0.0001	−4.02	4890.4	0.00006
**Determiners in PPs**	***b***	***se (b)***	***t***	***df***	***p***
Intercept	−6.92	0.039	−177.12	18.9	<0.00001
Context Frequency	0.0001	0.0001	2.08	4802.5	0.038
Predictability	−0.0006	0.001	−0.59	4793.8	0.56
Informativity	0.042	0.008	5.59	21.55	0.00001
Predictability:Context Frequency	−0.0001	0.00001	−5.39	4794.5	<0.00001
Informativity:Context Frequency	−0.0001	0.00002	−5.46	4802.8	<0.00001

The results in Figure 1 and Figure 2 are exactly as we would expect from adaptive partial pooling: predictability has the largest effect in frequent contexts, where it can be reliably estimated (see also [26]). In turn, informativity has the largest effect in rare contexts, where predictability estimates are least reliable. This is expected either because 1) our/researchers’ estimates of predictability are off in rare contexts, and are more accurate in frequent contexts, or 2) because speakers’ pronunciations are driven by predictability in the current context (or in-context memories of pronunciations) to the extent that the predictability estimate is reliable (or in-context pronunciation tokens are numerous). To put it another way, how much of the effect of informativity and its interaction with context frequency is an artifact of sampling noise in the data and how much is the result of the speakers’ adaptive behavior?

### 3.2. Simulating What Would Happen if There Were No Informativity Effect

As described in the Materials and Methods, we created 1000 simulated datasets in which there was no real effect of informativity, and no real correlation between the effect of predictability and context frequency, but the main effect of predictability, the distribution of context frequencies and the amount of noise were the same as in the real data. Predictability was generated by sampling from a binomial distribution, i.e., for each context, flipping a coin with probability corresponding to true predictability n times, and then noting the resulting sample predictability value.

For these simulations, I report the results for analyzing the data with both a mixed-effects model and a fixed-effects-only model because both analysis methods are common in the literature, and there is some discussion on whether adding a random effect of item makes sense for corpus data in which most words are observed only once (as is the case in the post-disfluency sample). Adding the interactions with context frequency reduces the false alarm rate (i.e., the likelihood of detecting a spurious but significant effect of informativity), as does adding a random intercept by word. However, as discussed below, the likelihood of a false alarm on informativity can nonetheless be high depending on the true size of the predictability effect: the larger the effect of predictability, the greater the likelihood of detecting a spurious effect of informativity.

**Figure 3:**
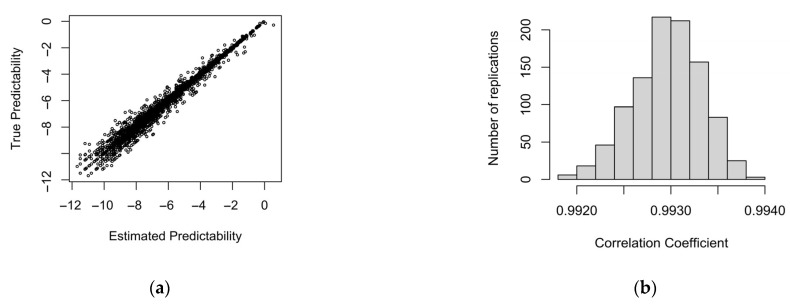
(**a**) True predictability vs. estimated predictability based on the sample generated by binomial sampling with probability proportional to true predictability. (**b**) A histogram of the correlation coefficients between true predictability and estimated predictability across the 10,000 replications.

Figure 3 shows that true predictability and sample predictability are highly correlated, with r > 0.99.

**Figure 4:**
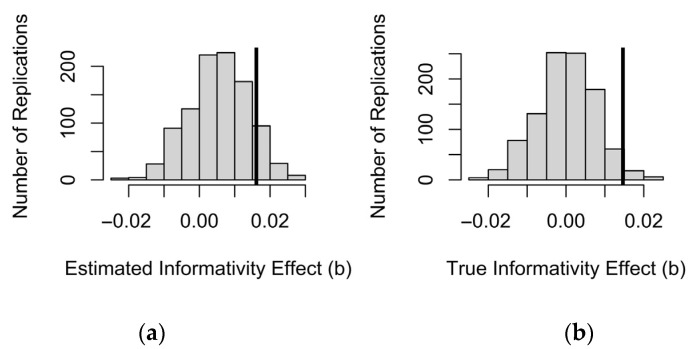
The distribution of informativity coefficients across 1000 replications of sampling from a population in which there is no real effect of informativity. In (**a**), predictability and informativity used to predict durations are estimated from the observed sample. In (**b**), true predictability and informativity are used. Coefficients to the right of the thick vertical lines are significant at the 0.05 level in the expected direction.

Figure 4 shows data generated from a fixed-effects-only model with only a main effect of predictability. The data are generated from true predictability × b(Pred) + noise in Table 1, with noise matching the standard deviation of the residuals, but estimated predictability and estimated informativity are then used as predictors in the analysis. Despite the strong correlation, the divergence between true predictability and sample predictability is sufficient for informativity to have a significant (but spurious) effect at the 0.05 alpha level more than 9% of the time when probability and informativity are estimated from the sample (Figure 4a). Furthermore, this spurious effect of informativity is usually in the same, theoretically motivated, direction as in the real data (73% of the coefficients in Figure 4a are positive and 91% of significant coefficients are). Of course, if this test were perfectly calibrated, then it should have come out significant by chance 5% of the time and be positive or negative about equally often. This is what we observe in Figure 4b, where the (unobtainable in practice) true predictability and informativity values are used as predictors instead of their values in the simulated data sample. Thus, the small amount of sampling noise seen in Figure 3 is enough to double the false alarm rate, if we simply accept that an observed effect of informativity is a true effect as long as it is significant in a regression model that also includes predictability.

**Figure 5:**
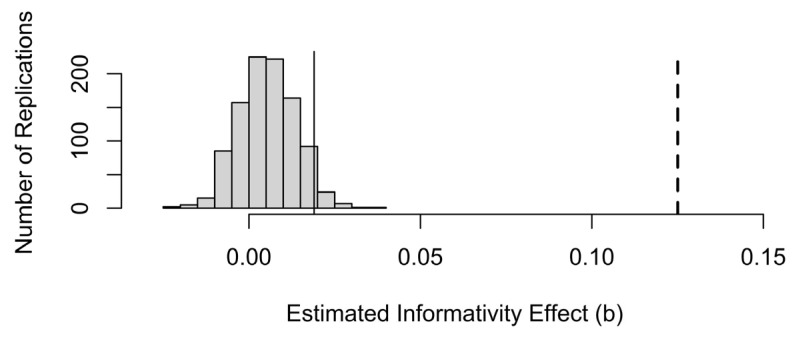
The distribution of informativity coefficients from Figure 4a relative to the informativity coefficient in the real data, shown by the dashed vertical line. The data-generating process behind Figure 3a has a 5% chance of generating a coefficient for informativity to the right of the thin solid vertical line.

However, Figure 5 shows that the observed size of the informativity coefficient, shown by the vertical dashed line, is much larger than the coefficients obtained in the simulated samples, where there is no true effect of informativity. Thus, although a significant effect of informativity could be spurious, an effect of the size we observed is unlikely to be due to sampling noise alone. Since we made 1000 simulations, *p* < 1/1000 or 0.001.

**Figure 6:**
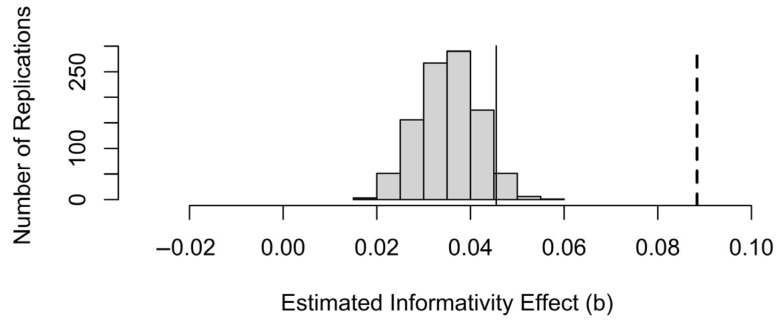
The distribution of informativity coefficients generated from a mixed-effects model in which there is no effect of informativity but there is the same coefficient for predictability and the same random effect of word as in the real data. The data-generating process has a 5% chance of generating a coefficient for informativity to the right of the thin solid vertical line. The dashed vertical line shows the informativity coefficient from the model of real data in Table 2.

A mixed-effects model with a main effect of predictability and a random effect of word will attribute some of the variance attributed to informativity in Table 2 to the random effect of word instead. This is the part of the informativity effect that is attributable to uncontrolled differences between words. Therefore, when the data to be fit are generated from a mixed-effects model instead of the fixed-effects-only model in Figure 5, there will be an elevated chance of finding a spurious effect of informativity in these data even if “true informativity” is used as a predictor, i.e., the equivalent of Figure 4b would still show a >2.5% chance of informativity effect in the expected direction. However, this combined effect should still be smaller than the effect of informativity observed in Table 2. Figure 6 demonstrates that the effect of informativity in the mixed-effects model is closer to the distribution of simulated informativity effects and that the simulated informativity coefficients are always positive (informative words being longer). Indeed, about a third of the informativity effect in the data is attributable to the random effect of word identity and the correlation of informativity with predictability. However, the coefficient in the real data is still larger than in the simulated data, suggesting that some of the effect of informativity in Table 3 is real. The generating model can of course be made more complex, and realistic, by including additional predictors beyond the random intercept of word, as in [1,2,3,4,5,6,7,8,9,10,11], which may change this conclusion.

The size and likelihood of a false detection of the informativity effect depend on the size of the predictability effect, and the model structure, as shown in Table 4. The likelihood of a false positive on informativity increases as the effect of predictability grows. Informativity captures some of the effect of true predictability, which is not captured by estimated predictability because of the sampling noise causing true and estimated predictability to diverge. The larger the effect of true predictability, the more variance in duration is left over for informativity to capture, and therefore the greater the likelihood that the spurious effect of informativity will be large enough to be significant. Unsurprisingly, the likelihood of a false alarm decreases when interactions with context frequency or the random effect of word are included.

### 3.3. Interactions with Context Frequency

So far, we have examined whether the main effect of informativity in Table 1 and Table 2 could be spurious. What of the predictability:context.frequency and informativity:context.frequency interactions in Table 3? How likely are they to be spurious, and to what extent are these interactions due to partial pooling helping address sampling noise in predictability? For simplicity, all simulations in this section assume that there is a real random effect of word in the data, because this is the standard assumption in the literature. However, we vary whether the model applied to the dataset includes the random effect of word (a random intercept).

Table 5 shows that, when the random effect of word is in the model, the likelihood of a false alarm on the interactions with context frequency decreases when there is a true main effect of informativity. This is not the case if the random intercept for words is omitted. When the random effect of word is (falsely) omitted, spurious interactions between informativity and context frequency are almost always detected when there is a main effect of informativity.

The likelihood of a spurious interaction between predictability and context frequency increases with increasing size of the predictability effect: the larger the main effect of predictability, the larger the difference in apparent effect size between frequent and rare contexts. The random effect of word effectively keeps this false alarm rate on interactions between predictability and context frequency under control. The presence of a true informativity effect reduces the likelihood of finding a spurious interaction between predictability and context frequency.

Overall, however, we can note that there is a high risk of an interaction between context frequency and informativity such that the informativity effect increases in rare contexts being spurious, due simply to noise in predictability estimates. All numbers in the right column are above the nominal level of 0.025.

Since both the spurious effect of informativity and its interaction with context frequency are attributable to partial pooling, the two effects should show up together in our simulations. This is shown in Table 6 for the mixed-effects model: when we detect a spurious informativity effect, we find that it gets smaller in frequent contexts 89–99% of the time, compared to the chance level of 50%. When a significant spurious interaction in this direction is detected (the informativity effect reduces in frequent contexts), a spurious simple effect is always detected as well. Thus, a spurious interaction with context frequency goes together with a spurious main effect of informativity. When informativity simply helps explain the variance that is really due to predictability the effect of informativity will almost always decrease in frequent contexts.

**Figure 7:**
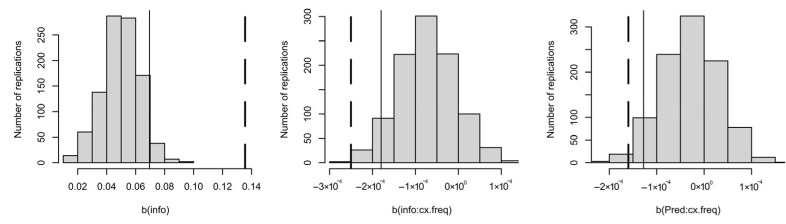
Fitting the full model in Table 3 to data generated without any true effect of informativity and no speaker/corpus probability mismatch. Dashed lines show observed coefficients from Table 3. Thin solid lines show the critical value of the coefficient in the same direction. Simple effect of informativity (**left**), and interactions of context frequency with informativity (**middle**) and predictability (**right**).

However, as shown in Figure 7, the simple effect of informativity is stronger than expected if all of the variance shared between informativity and predictability were attributable to predictability. Thus, again some of the informativity effect appears to not be spurious. For the interactions, there is more uncertainty that they are not due simply to predictability estimates being noisier in rare contexts. The interactions are significant at the 0.05 level (as shown by the fact the dashed line is farther from the middle of the distribution than the solid line in the middle and right panels). However, the interactions are not significant at the 0.001 level, even though *p* < 0.0001 in Table 3. This is because a spurious effect of informativity in this direction tends to come with interactions in this direction. That is, when the data are generated by a constant predictability effect that is independent of context frequency, a spurious informativity effect will appear to increase in rare contexts while the predictability effect will appear to decrease in rare contexts. The simulations in Figure 7 allow one to estimate whether this is a plausible explanation for the main effect of informativity and the interactions between predictability or informativity with context frequency observed in one’s dataset.

## 4. Discussion

Informative words have been repeatedly observed to be longer than less informative words. Furthermore, the effect of informativity has been observed to hold even if predictability given the local context is included in the regression model of word duration [1,2,3,4,5,6,7,8,9,10,11]. The present paper pointed out that informativity could improve local predictability estimates because of sampling noise in the latter, especially in rare contexts. This means that one would expect some effect of informativity to come out in a regression model that includes predictability even if reduction were driven by predictability alone. The same reasoning predicts that informativity effects should be stronger in rare contexts where the context-specific predictability estimates are noisiest. We showed this to be the case in two datasets at the opposite ends of the predictability, informativity and duration continua.

We then examined by simulation whether an effect of informativity observed in a real dataset is small enough to have emerged from sampling noise, in a world where differences in word duration were entirely due to predictability in the local context. We have shown that there is bias in informativity coefficient estimates: a spurious effect of informativity does come out significant and in the expected direction (showing an effect in the opposite direction of the reductive effect of predictability) more often than the *p* value in a regression model that includes both predictability and informativity would lead us to expect. This is also true for the interactions between context frequency and either predictability or informativity. However, the effect of informativity is smaller than the one observed in the real data. Therefore, we can conclude that the effect of informativity in the real data is unlikely to be entirely attributable to sampling noise in predictability estimates.

We showed that predictability and informativity interact with context frequency as one would expect from the predictability estimates being less reliable when they are conditioned on rare contexts. The interaction of predictability and context frequency has previously been observed in [26], whereas the interaction of informativity with context frequency is a new finding. This finding is predicted by the idea of informativity effects being due to adaptive partial pooling of probabilities across contexts. Our simulations show that the interaction of predictability with context frequency observed in the real data might be attributable to this effect of the Law of Large Numbers (i.e., *p* > 0.05). However, some of the interaction of informativity with context frequency may not be attributable to there being more noise in predictability estimates for informativity to compensate for in rare contexts. So, overall, not all of the effect of informativity is attributable to predictability, and the effect of informativity may genuinely be stronger in rare contexts.

This result extends prior simulation work in [24] that argued that the segmental informativity effect observed in [32] (e.g., [t] is less informative and more likely to be deleted than [k] in English) could not be attributed to noise in local predictability estimates. Like [24], we observe that most of the effect of informativity in the real data is not entirely due to noise in predictability estimates. However, this result is more surprising on the lexical level examined here than on the segmental level examined in [24], because (as pointed out there) segments are abundantly frequent and few in number, and therefore sampling noise in predictability is rather low at the segmental level. In contrast, word frequency distributions are Zipfian, with half the words occurring only once in the corpus, resulting in many contexts where estimating local predictability is virtually impossible. This concern was previously acknowledged in [24], which reported also running simulations at the lexical level, but this is the first time lexical simulation results are reported in the literature.

Unlike [24], which simulated how strongly true and estimated predictability and informativity values correlate depending on sample size, we took the approach of simulating the dependent variable, duration. This is likely because [24] focused on early studies that included only predictability or only informativity in their models, whereas we focus on the studies that included both predictors in the same model [1,2,3,4,5,6,7,8,9,10,11]. In the context of a model that includes both predictability and informativity, as in [1,2,3,4,5,6,7,8,9,10,11], a strong correlation between informativity and predictability is not a guarantee that an effect of predictability would often be falsely attributed to informativity. For example, in the post-disfluency dataset, informativity and predictability correlate at |*r*| > 0.91. Nonetheless, a true effect of predictability is seldom falsely attributed to informativity in its entirety—predictability is almost always significant in our simulations: despite the strong correlation, the sample size is large enough to detect a real effect of predictability with informativity in the model.

The advantage of simulating duration is also that we can determine how large an effect of informativity is expected to be if it were entirely spurious, and predictability had the same bivariate relationship with duration as in the real data. This is important because the likelihood of observing a spurious informativity effect and its size depend strongly on the size of the predictability effect on the dependent variable. Furthermore, by simulating the dependent variable we are able to estimate whether all of the observed informativity effect is likely to be attributable to noise in the predictability estimates.

Of course, showing that an informativity effect cannot be explained away by local predictability or by random differences between words leaves open the possibility that other stable characteristics of words that do not change across contexts and correlate with informativity would explain it away. We do not consider these alternatives here because such alternative explanations are adequately addressed by standard multiple regression methods, as in [1,2,3,4,5,6,7,8,9,10,11]. However, the present simulations show that some of the effect of informativity in these previous studies could be due to noise in predictability estimates. This raises the possibility that the remainder of the informativity effect might still be explained away by other stable, context-independent but word-specific factors (e.g., [44]). The methodology proposed here can incorporate such additional predictors, by creating simulated data in which all predictors but informativity have the effects they do in the real data, fitting a model with all predictors, including informativity, and testing how often we obtain an informativity effect of the observed magnitude and direction. A central point of this paper is that this remains an important direction for future work: the collinearity between informativity and predictability means that the effect of informativity is on looser ground than it appears to be.

The simulations above assume that corpus frequencies and probabilities are unbiased estimators of the probabilities (or activations, in a neural network framework) that drive reductions. While this is the standard simplifying assumption of all corpus studies, it may not be justified; e.g., [45]. For example, words of zero frequency in a large corpus nonetheless vary in the probability of a speaker knowing each word, and these probability differences affect lexical processing variables like reaction time in lexical decision [46] and likely also affect production measures like duration. The present simulations assume that words of frequency zero in the corpus have a zero probability of occurrence, which is of course not true for many such words. It does not appear to be a dangerous assumption because we do not have duration measurements for such words in any case so they would not be able to influence our estimate of the relationships between duration, predictability and informativity. However, the general possibility that the mismatch between predictability in a corpus and predictability in a speaker’s experience might distort corpus predictability estimates for rare items and in rare contexts beyond simple sampling noise means that we might be underestimating noise in predictability estimates in such situations. If so, we may need to be particularly cautious in arguing that informativity effects found with such materials cannot be due to predictability.

However, let us assume that some of an effect of informativity is genuine. How then should we interpret it? Here, the notion of adaptive partial pooling also provides a novel perspective. A genuine effect of informativity and its interaction with context frequency can be due to speakers themselves representing their linguistic experience in a way that results in adaptive partial pooling, as argued in [17,47]. Adaptive partial pooling is directly predicted by probabilistic models of the mind [17,47,48], which propose that the language learner engages in hierarchical inference, building rich structured representations and apportioning credit for the observations among them. However, it is also compatible with other “rich memory” models in which experienced observations are not discarded even if the learner generalizes over them. All such rich memory models can be understood as performing adaptive partial pooling at the time of production. For example, an exemplar/instance-based model stores specific production exemplars/instances tagged with contextual features [49,50]. It then produces a weighted average of the stored exemplars, with the weight reflecting overlap between the tags associated with each exemplar and the current context. The average is naturally dominated by exemplars that match the context exactly when such exemplars are numerous and falls back on more distant exemplars when nearby exemplars are few. Modern deep neural networks also appear to be rich memory systems that implement a kind of hierarchical inference when generating text, in that best performance is achieved when the network is more than big enough to memorize the entire training corpus [51,52]. Increasing the size of the network beyond the point at which it can memorize everything appears to benefit the network specifically when the context frequency distribution is Zipfian [53,54], by allowing it to retrieve memorized examples when the context is familiar and to fall back on generalizing over similar contexts when it is not. The existence of implicit partial pooling in such models may be why attempts to add explicit partial pooling to them to improve generalization to novel contexts [55] have led to limited improvements in performance.

Within the usage-based phonology literature, informativity effects have been considered to be one of a number of effects that go by the name of “frequency in favorable contexts” (FFC). In general, the larger the proportion of a word’s tokens that occur in contexts that favor reduction, the more it will be reduced in other contexts [18,19,20,21,22,23]. Informative words can be seen as one type of low-FFC word, because (by definition of informativity) they tend to have low predictability given the contexts in which they occur. In this literature, informativity effects have been argued to be due to storage of phonetically detailed pronunciation tokens in the memory representations of words [18,19] and are therefore often thought to be categorically distinct from the online, in-the-moment reduction effects driven by predictability in the current context.

However, the distinction between informativity and local predictability may also be an artifact of how we traditionally operationalize the notion of “current context”. In reality, a word is never in exactly the same context twice. All contexts have a frequency of 1 if you look closely enough, and predictability of a word in a context always involves some generalization over contexts. That is, true predictability must always lie between predictability given the current context and average predictability across contexts (i.e., the inverse of informativity). The present paper treated contexts as being the same if they end in the same word. This is motivated by findings that the width of the context window does not appear to matter for predicting word duration (and other measures of word accessibility) from contextual predictability [35,56]. That is, language models with very narrow (one-word) and very broad windows generate upcoming word probabilities that are equally good at predicting word duration and fluency [35,56,57], suggesting that it is largely predictability given the preceding word that matters. However, [56] nonetheless shows that a large language model (with a short context window) outperforms an n-gram model in yielding predictability estimates predictive of word durations. It therefore seems likely that the notion of “current context” in our studies is too narrow and speakers actually generalize over the contexts that we considered distinct. For example, even if it is only the preceding word that matters, distributionally similar preceding words might behave alike, which a large language model would pick up on by using distributed semantics word representations (also known as embeddings) instead of the local codes of an n-gram model. Thus, another direction for future work is to try to determine what makes contexts alike in conditioning reduction.

## Figures and Tables

**Table 4:** Effect sizes and false alarm rates for a spurious effect of informativity (probability of detecting a significant informativity effect at the 5% level in the expected direction when its true effect is zero). The proportions parentheses would be 2.5% (5%/2) if the probability of a false alarm were truly 5%. True model consists of a main effect of predictability (Pred) and a random intercept for word.

Size of the True Predictability Effect	Model	Mean Spurious Effect of Informativity (Probability of a Spurious but Significant Result)
–0.04	Pred + Info	0.05 (100%)
	(Pred + Info)*CxFreq	0.084 (85%)
	Pred + Info + (1|Word)	0.024 (96%)
	(Pred + Info)*CxFreq + (1|Word)	0.036 (82%)
–0.08	Pred + Info	0.10 (100%)
(as in Table 2)	(Pred + Info)*CxFreq	0.17 (100%)
	Pred + Info + (1|Word)	0.035 (99.8%)
	(Pred + Info)*CxFreq + (1|Word)	0.05 (96%)

**Table 5:** The probability of false alarms in fixed-effects-only and mixed-effects models that include interactions with context frequency, on the effect of informativity and the interactions with context. The dataset has a true effect of predictability (varying in size as shown) and a true random intercept of word (of a constant size across the rows) but no true effect of informativity and no true interactions with context frequency. Nominal false alarm level = 0.025.

Model	*b* (Pred)	*b* (Info)	False Alarm or Power on Info	False Alarm on Pred:CxFreq	False Alarm on Info:CxFreqs
Fixed	−0.02	0	0.94	0.06	0.32
	−0.08	0	1	0.50	1
	−0.02	0.044	1	0.14	0.99
	−0.08	0.044	1	0.36	1
Mixed	−0.02	0	0.50	0.05	0.11
	−0.08	0	0.96	0.07	0.21
	−0.02	0.044	1	0.023	0.08
	−0.08	0.044	1	0.015	0.10

**Table 6:** The probability of detecting a spurious but significant effect of informativity in the expected direction (second column), the probability of the interaction between informativity and context frequency being in the expected direction given that a significant spurious main effect of informativity is detected (third column) and the probability of a main effect of informativity being in the expected direction given that the interaction is falsely detected (fourth column) as a function of the size of the true predictability effect.

*b* (Pred)	Probability of a False Alarm on Info	Probability of Info:CxFreq in the Expected Direction Given a False Alarm on Info	Probability of Info in the Expected Direction Given a False Alarm on Info:CxFreq
−0.02	0.50	0.99	1
−0.08	0.96	0.89	1

## Data Availability

Code for replicating the simulations and running your own is available at https://osf.io/c8aws, accessed on 4 July 2025.
